# Negative pressure wound therapy promotes muscle‐derived stem cell osteogenic differentiation through MAPK pathway

**DOI:** 10.1111/jcmm.13339

**Published:** 2017-09-25

**Authors:** Hong Liu, Xun Zheng, Liang Chen, Chao Jian, Xiang Hu, Yong Zhao, Zonghuan Li, Aixi Yu

**Affiliations:** ^1^ Department of Orthopedics Zhongnan Hospital of Wuhan University Wuhan Hubei China

**Keywords:** osteogenic differentiation, proliferation, muscle‐derived stem cells, negative pressure wound therapy

## Abstract

Negative pressure wound therapy (NPWT) has been revealed to be effective in the treatment of open fractures, although the underlying mechanism is not clear. This article aimed to investigate the effects of NPWT on muscle‐derived stem cell (MDSC) osteoblastic differentiation and the related potential mechanism. The cell proliferation rate was substantially increased in NPWT‐treated MDSCs in comparison with a static group for 3 days. There was no observable effect on the apoptosis of MDSC treated with NPWT compared with the control group for 3 days. The expression levels of HIF‐1α, BMP‐2, COL‐I, OST and OPN were increased on days 3, 7 and 14, but the expression level of Runx2 was increased on days 3 and 7 in the NPWT group. Pre‐treatment, the specific inhibitors were added into the MDSCs treated with NPWT and the control group. ALP activity and mineralization were reduced by inhibiting the ERK1/2, p38 and JNK pathways. The expression levels of Runx2, COL‐I, OST and OPN genes and proteins were also decreased using the specific MAPK pathway inhibitors on days 3, 7 and 14. There were no significant effects on the expression of BMP‐2 except on day 3. However, the expressions of the HIF‐1α gene and protein slightly increased when the JNK pathway was inhibited. Therefore, NPWT promotes the proliferation and osteogenic differentiation of MDSCs through the MAPK pathway.

## Introduction

NPWT is an effective treatment method of various complex wounds. High‐energy trauma, open fractures and excessive soft tissue damage are often seen in clinical work. There are plenty of studies that reported that NPWT could promote the growth of granulation tissue, reduce tissue oedema, increase wound blood supply topically and decrease the incidence of infection 1, 2, 3, 4.

One way that NPWT promotes wound healing is by creating a subatmospheric environment, acting at the level of the interstitium to eliminate unwanted oedema, inflammatory mediators and bacteria, and by removing the volume that obstructs the inflow and out‐flow, thereby allowing greater nutrient and oxygen inflow as well as venous drainage 5, 6. Furthermore, the mechanical strain allows microdeformation and stretch at the cellular level, permitting cellular chemotaxis, angiogenesis and new tissue formation 6. Numerous reports have documented that NPWT could successfully promote wound healing and has no harmful effect on fracture healing 7, 8, 9, 10, but the advantages of NPWT on bone treatment remain under debate.

MDSCs are a type of stem cells that have a characteristic of self‐renewal and differentiation capacity. Liu *et al*. 11 reported that MDSCs were recognized as one of the key cells during open fracture healing. They found that the contribution of MDSCs to the healing of callus tissues was insignificant in closed tibia fractures. However, approximately 40% of the cells in an open fracture with periosteal stripping were MDSCs. MDSCs show great osteogenic tendency induced with bone morphogenetic protein 2 (BMP‐2) or BMP‐4 12, 13, 14. The mitogen‐activated protein kinase (MAPK) signalling pathway, which included extracellular‐regulated kinase 1/2 (ERK1/2), p38 MAPK and c‐Jun N‐terminal kinase (JNK), occupied a central role in osteogenic differentiation. Payne *et al*. 15 described that MDSCs could differentiate into osteoblasts *via* ERK1/2 and p38 in the induction of BMP2. Guicheux *et al*. 16 reported that the p38 and JNK pathways participated in BMP‐2‐induced osteoclast differentiation. Our previous study found that NPWT could promote periosteum‐derived mesenchymal stem cells (P‐MSCs) proliferation and osteogenic differentiation 17. Therefore, we suggest that NPWT‐promoted open fracture healing might be related to osteogenic differentiation of MDSCs. However, whether proliferation and osteogenic differentiation of MDSCs are under continuous negative suction has not been reported.

In this study, we illustrate that NPWT promotes MDSC proliferation and osteogenic differentiation and investigate the underlying mechanism. Therefore, we discovered that NPWT could promote MDSC proliferation through cell counting kit‐8 (CCK‐8) analysis, but there were no obvious effects on apoptosis. NPWT could promote MDSC osteogenic differentiation by analysis of alkaline phosphatase (ALP) activity, alizarin red staining and expression of osteoblast‐related genes and proteins. Moreover, ALP activity, mineralization, expressions of osteoblast‐related genes and proteins were decreased when the ERK1/2, p38 and JNK pathways were inhibited. Therefore, we reveal that NPWT could promote MDSC osteogenic differentiation through the MAPK pathway.

## Materials and methods

### Isolation and purification of MDSCs

In this study, we used a modified pre‐plate technique to obtain MDSCs from 3‐week‐old Sprague Dawley (SD) rats (Laboratory Animal Center of Wuhan University, China) 18. Briefly, the isolated cell was removed from the gastrocnemius and then suspended in the proliferation media (10% foetal bovine serum (FBS, Gibco, Carlsbad, CA, USA), 10% horse serum (HyClone, South Logan, UT, USA), 0.5% chick embryo extract (GEMINI, West Sacramento, CA, USA) and 1% penicillin–streptomycin (P/S, Invitrogen, Waltham, MA, USA) were added to low‐glucose Dulbecco's modified Eagle's medium (L‐DMEM, HyClone)).

The adherent cells, which were considered as pre‐plate 1 (PP1), were cultured in a humidified atmosphere with 5% CO2 at 37°C, and the non‐adherent cells were considered as pre‐plate 2 (PP2). Following this step, the cells were successively identified until PP6. MDSCs were used for the following experiments from the third passages. MDSCs were cultured as formerly described 15, 16; osteogenic medium (OSM) (50 mg/ml ascorbic acid, 10 mM β‐glycerophosphate and 0.1 mM dexamethasone (all from Sigma‐Aldrich, St. Louis, MO, USA) and with BMP‐2 (100 ng/ml) (Syagen, Santa Clara, CA, USA)) was supplemented with L‐DMEM (HyClone)). According to a previous study 15, 16, we selected PD98059 (25 μM), SB203580 (10 μM) or SP600125 (25 μM) to inhibit the ERK1/2, p38 and JNK pathways, respectively. These special inhibitors were purchased from the Cell Signaling Technology (CST, Danvers, MA, USA). The cell clots were incubated with these inhibitors for 24 hrs before MDSCs treated with or without NPWT.

### Preparation of NPWT bioreactor

We assembled the bioreactor according to previous studies 17, 19. In short, moderate size foam (VSD Medical Technology Inc., Wuhan, China) was placed above the prepared cell matrix containing 2 × 10^4^ MDSCs. A drape was used on the top of the well to ensure the well was sealed. A scalp needle passed through the 3 M bumpon into the foam and then connected with a vacuum negative pressure pump (VSD Medical Technology Inc.). The pressure value of negative pressure pump was set as uninterrupted suction at −125 mmHg. Another needle was passed through the O‐ring elastomeric disc and arrived at the bottom of plate. This needle was connected with a peristaltic pump (Longer, Baoding, China), which injected OSM at seven ml per 24 hrs per well. The static group, which has no vacuum suction, was cultured in the same bioreactor. These groups were cultured in the CO2 incubator with 5% CO2 at 37°C.

### Flow cytometry analysis

We used flow cytometry to identify MDSCs. PP6 cells were washed with phosphate‐buffered saline (PBS, HyClone) two times, digested with 0.25% trypsin (HyClone) and suspended. The suspended cells were collected in tubes, centrifuged and suspended once sequentially. Each tube received FITC‐conjugated CD45 (Abcam, Cambridge, UK), FITC‐conjugated CD34 (Abcam), FITC‐conjugated Sca‐1 (Abcam) and FITC‐conjugated Desmin (Abcam). The analysis was performed by a flow cytometer (Becton Dickinson, Franklin Lakes, NJ, USA) after several centrifugations and suspensions.

### CCK‐8 assay

The cell clots were treated with NPWT or static conditions for 3 days. Fresh medium containing a CCK‐8 (Dojindo, Kumamoto, Japan) was added to these cells, transferred to 96‐well plates and then measured on a microplate reader (DR‐200Bs, Beijing, China) with absorbance at 450 nm after incubation at 37°C for 3 hrs.

### TUNEL assay

MDSCs were cultured with NPWT or static conditions for 3 days. These cells were fixed with 4% paraformaldehyde for 30 min., washed with PBS (HyClone, three times, 5 min. each) and then permeabilized in 0.5% Triton X‐100 for 2 min. Then, the freshly prepared TUNEL reaction mixture (50 μl TdT and 450 μl dUTP) was added for 60 min. The cell nuclei were stained by DAPI for 5 min. Then, we used a confocal microscope (LSM710, ZEISS, Jena, Germany) to visualize and photograph the cells. The apoptosis rate was calculated according to the number of TUNEL^+^ cells divided by the randomly selected region total number of cells.

### Matrix mineralization

We used alizarin red staining to demonstrate mineralization of the clot cells on days 3, 7 and 14. The clots were incubated with 2% alizarin red S (Sigma‐Aldrich) for half an hour, washed with PBS (HyClone) and then imaged using an Olympus Inverted Microscope (Olympus, Tokyo, Japan). Alizarin red staining was quantified using the areas and integral optical density (IOD).

### ALP activity assay

ALP activity was detected by nitrobenzene phosphate method. Briefly, the cells were lysed on days 3, 7 and 14 of treatment with or without NPWT. Lysates were incubated with pNPP (Sigma‐Aldrich) solution for 15 min. in the culture conditions, and the reactions were then stopped by adding NaOH. Subsequently, the ALP activity was determined by measuring the OD values for absorbance at 405 nm and expressed using nmol pNPP/μg of total cellular protein.

### Quantitative real‐time PCR analysis

The real‐time fluorescent quantitative PCR (RT‐PCR) was performed according to a previously reported protocol 17. Briefly, the total RNA was extracted on days 3, 7 and 14 of treatment with or without NPWT using TRIzol reagent (Invitrogen). The first strand of cDNA was obtained from the total RNA using oligo‐dT primers and reverse transcriptase (Takara Bio, Kusatsu, Shiga, Japan). RT‐PCR was completed in the StepOne Real‐Time PCR instrument (Life Technologies, Waltham, MA, USA). GAPDH mRNA expression was used as an endogenous control. The fold changes were calculated according to the manufacturer's instructions (Takara Bio). The PCR contained a first step of denaturation at 95°C for 1 min., followed by total 40 cycles at 95°C for 15 sec., 58°C for 20 sec. and 72°C for 45 sec., followed by measurement using the 2−ΔΔCt method. The primer sequences used in the present study are listed in Table. 1.

**Table 1:** The primer sequences for each primer used in the RT‐PCR

Genes	Forward primer(5′‐ 3′)	Reverse primer(5′‐ 3′)
GAPDH	CGCTAACATCAAATGGGGTG	TTGCTGACAATCTTGAGGGAG
HIF‐1α	AAGCCCAGAGTCACTGGGACT	GTACTCACTGGGACTGTTAGGCTC
BMP‐2	GAAGCCATCGAGGAACTTTCAG	GGAAATTTTGAGCTGGCTGTG
Runx2	ACTCTGCCGAGCTACGAAATG	GGGACCGTCCACTGTCACT
COL‐I	CCGTGACCTCAAGATGTGCC	GAACCTTCGCTTCCATACTCG
OST	GATGCGGTCCCTAGTTCTACC	CCTCCAGTGAGTGGGATGTTT
OPN	AGCACACAAGCAGACGTTTTG	GCAACTGGGATGACCTTGATAG
ERK1	CTGGCTTTCTGACCGAGTATGT	AATTTAGGTCCTCTTGGGATGG
ERK2	GCACCAACCATTGAGCAGAT	TCACGGTGCAGAACATTAGCT
p38	AGATGCCGAAGATGAACTTCG	GGTCAGGCTCTTCCATTCGT
JNK	TCCAGCACCCGTACATCAAC	TCTTAGTTCGCTCCTCCAAATC

### Western blot analysis

We obtained the total protein from MDSCs treated with NPWT or static conditions or NPWT‐treated MDSCs with MAPK pathway‐specific inhibitors on days 3, 7 and 14 using a Total Protein Extraction Kit (Aspen, Wuhan, China). Equal amounts of protein obtained from the cell lysate were loaded onto 5% SDS polyacrylamide gel (Aspen) and transferred to polyvinylidene fluoride (PVDF) membranes (Millipore, Darmstadt, Germany). The membranes were obstructed with 5% BSA in TBS and then incubated overnight at 4°C with the following primary antibodies: rabbit anti‐GAPDH antibody (1:10000) (Abcam), rabbit anti‐RUNX2 antibody (1:1000) (Abcam), rabbit anti‐osteopontin (OPN) antibody (1:1000) (Proteintech Group, Inc, Chicago, IL, USA), rabbit anti‐osteonectin (OST) antibody (1:1000) (NOVUS, Littleton, CO, USA), mouse anti‐Collagen antibody (1:1000) (Abcam), mouse anti‐hypoxia‐inducible factor‐1α (HIF‐1α) antibody (1:500) (Santa, Dallas, Texas, USA), rabbit anti‐BMP2 antibody (1:2000) (Proteintech Group, Inc), rabbit anti‐ERK antibody (1:2000) (CST), rabbit anti‐P‐ERK antibody (1:1500) (CST), rabbit anti‐P38 antibody (1:2000) (CST), rabbit anti‐P‐P38 antibody (1:2000) (CST), rabbit anti‐JNK antibody (1:2000) (CST) and rabbit anti‐P‐JNK antibody (1:1000) (CST). Then, an HRP‐conjugated secondary antibody was used (1:10,000) (KPL, Milford, MA, USA). The protein bands were detected by the Immobilon Western Chemiluminescent HRP Substrate system (Millipore).

### Statistical analysis

We used the mean ± S.D. to express all of the values. The two groups were compared using a Student's unpaired *t*‐test. The statistical significance of the comparisons between the multiple groups was determined using an anova test. All tests were carried out using SPSS, v.18.0 (SPSS Inc., Chicago, IL, USA). We defined *P*‐values < 0.05 as statistically significant.

## Results

### Characterization of stem cell surface markers of MDSCs

**Figure 1:**
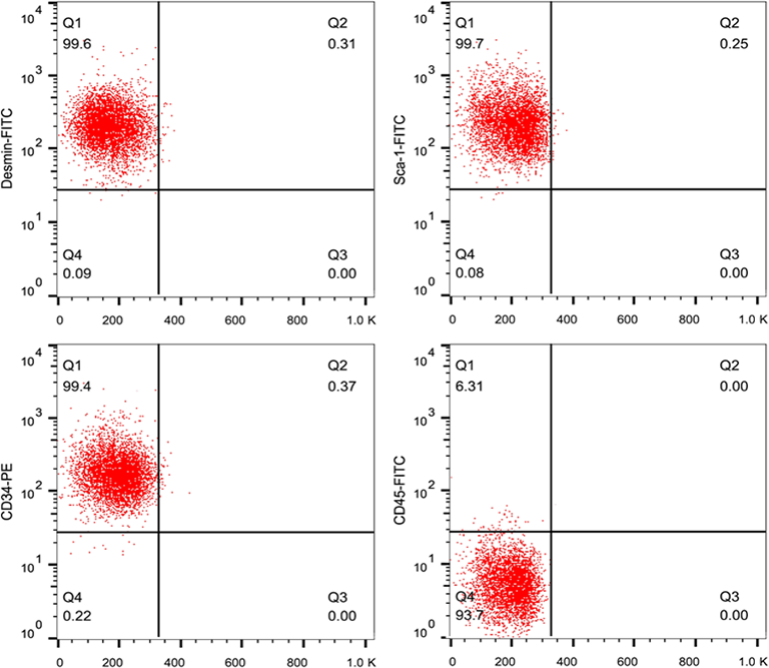
Characterization of MDSCs. Flow cytometry results of MDSCs at passage 6. The pp6 cells mainly expressed the surface markers Desmin, Sca‐1 and CD34; there was almost no expression of CD45.

We examined the cells isolated from the gastrocnemius muscle by the pre‐plate method 18. The PP6 cells are small, round and scattered with a ray. As shown in Figure 1, the flow cytometry analysis showed that the cultured cells expressed Desmin (99.6%), Sca‐1 (99.7%) and CD34 (99.4%) and almost no expression of CD45 (6.31%), which is consistent with the results of previous studies 12, 20. These results demonstrated that the PP6 cells were MDSCs, and the PP6 cells were used for subsequent experiments.

### NPWT promotes MDSC proliferation

**Figure 2:**
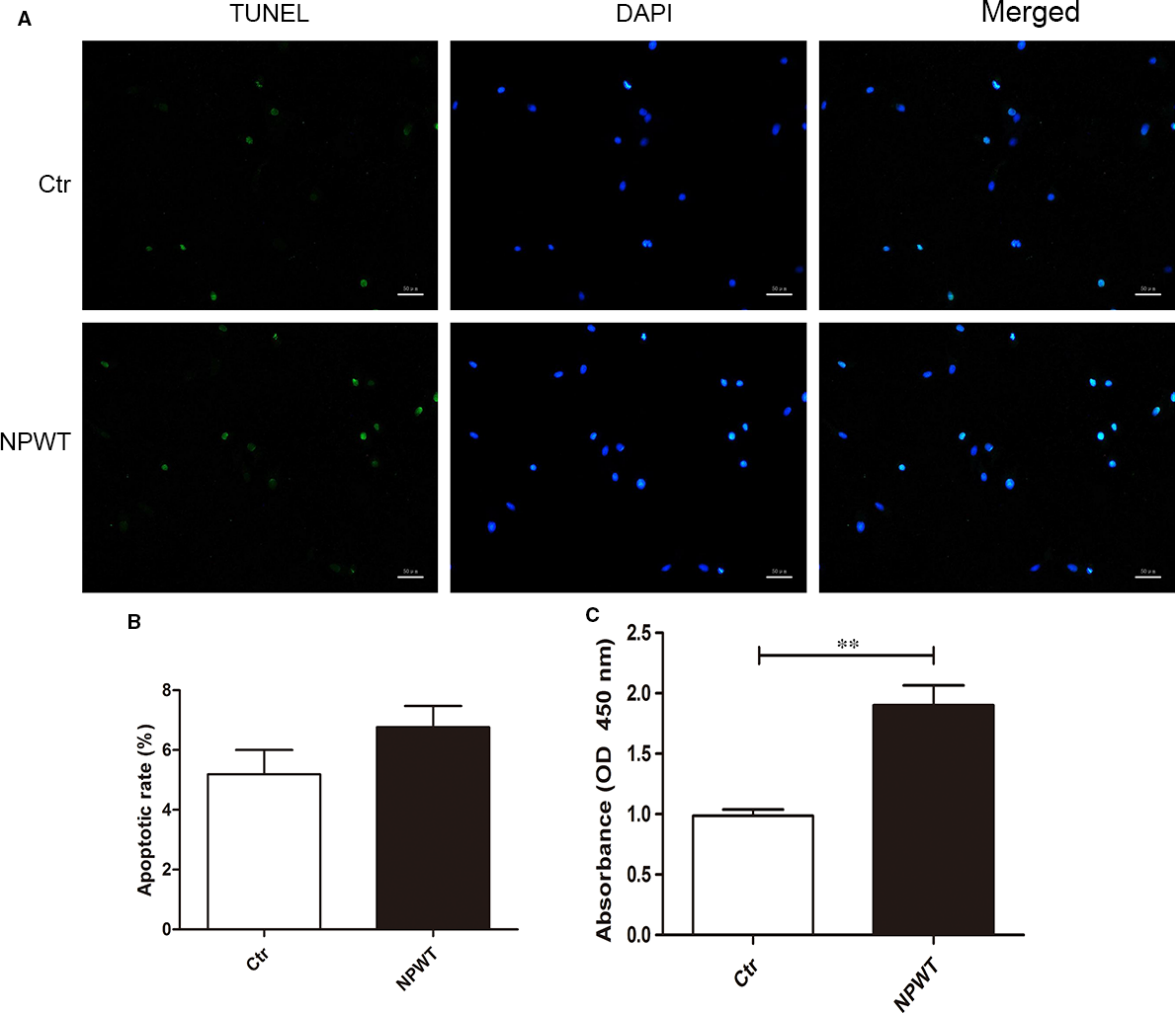
Cell proliferation and apoptosis assay**.** During 3 days of treatment with NPWT, apoptosis of MDSCs was evaluated by TUNEL assay. The images of TUNEL ^+^ cells (green fluorescence) are shown in (**A**, bars = 50 μm), and percentages of apoptosis cells in clots are shown in (**B**). Compared with the control group, 3 days of NPWT did not result in a significant increase in cell apoptosis. (**C**) The cell viability was measured on 3 days by a CCK‐8 assay, and optical density values at 450 nm were measured. Compared with control cells, NPWT‐treated cells had a higher OD value (**P <* *0.01).

The CCK‐8 assay was performed to determine the proliferation effect of NPWT on MDSCs. The data showed that the proliferation of MDSCs increased significantly when treated with NPWT for 3 days compared with control (Fig. 2C). Moreover, TUNEL analysis showed that the apoptosis rate has no obvious effect on MDSCs treated with NPWT for 3 days compared with the control group (Fig. 2A and B). The results revealed that NPWT could promote the proliferation of MDSCs.

### NPWT promotes MDSC osteogenic differentiation

**Figure 3:**
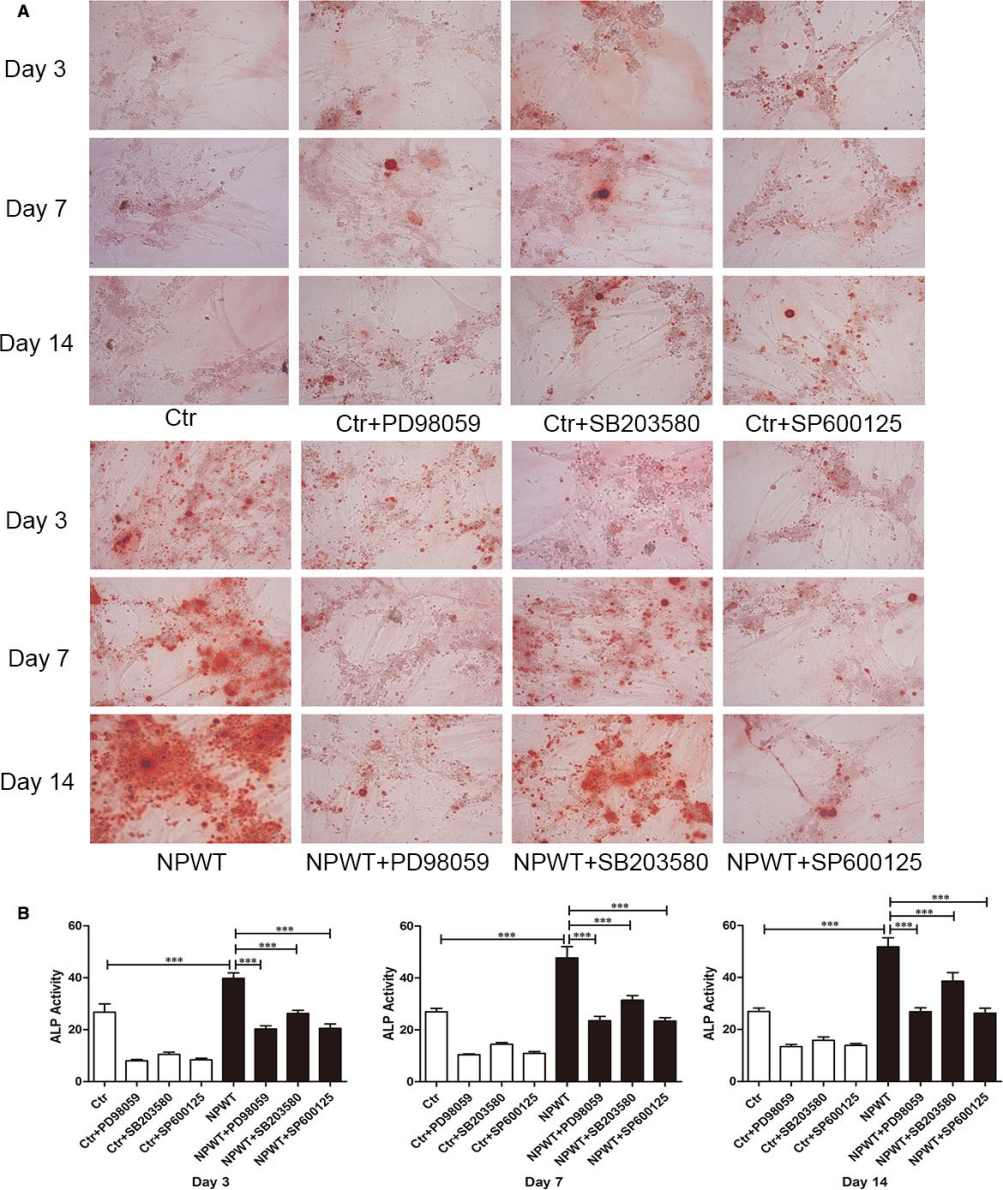
Alizarin red S staining and expression of ALP activity. (**A**) Images of alizarin red S staining of MDSCs treated with NPWT and inhibitors to the ERK1/2, p38 and JNK pathways on days 3, 7 and 14 (magnification: 200 × ). (**B**) ALP activity of MDSCs treated with NPWT and inhibitors of the ERK1/2, p38 and JNK pathways on days 3, 7 and 14. Inhibition of the ERK1/2, p38 and JNK pathways led to a decrease in ALP activity in MDSCs that were treated with NPWT. (***P <* *0.001).

The activities of ALP, alizarin red staining and expression levels of the osteogenic genes and proteins were measured to assess the effect of NPWT treatment on MDSC differentiation. There were extremely rare mineralization nodules under static conditions, but these nodules could be seen in the NPWT group. The nodules were big and dense in the NPWT group on days 7 and 14 (Fig. 3A).

The ALP activity substantially increased in the MDSCs treated with NPWT compared with controls; this activity was in accordance with the alizarin red staining results. The ALP activity analysis showed noteworthy increases in the NPWT group compared to the control group (Fig. 3B).

**Figure 4:**
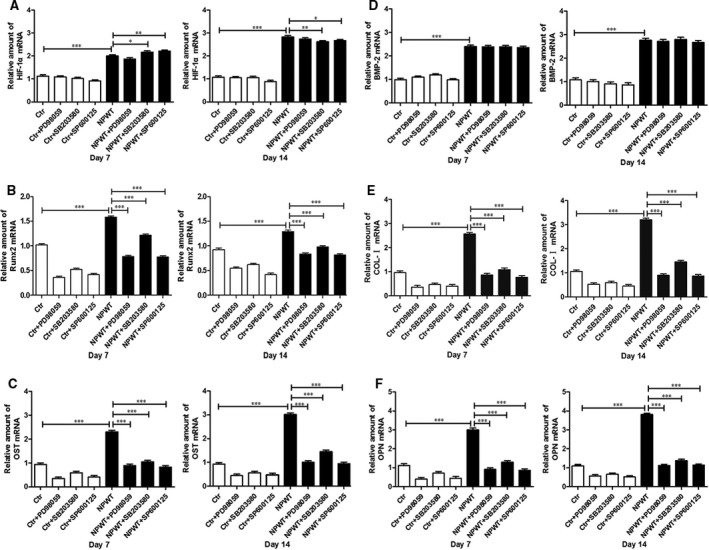
RT‐PCR analysis. HIF‐1α, BMP‐2, Runx2, COL‐I, OST and OPN gene expression in MDSCs treated with NPWT on day 7 and 14 incubation in the presence of the inhibitors of the ERK1/2, p38 and JNK pathways. The expressions of the specific genes (**A**) HIF‐1α, (**B**) Runx2, (**C**) OST, (**D**) BMP‐2, (**E**) COL‐I and (**F**) OPN were higher in the NPWT group than in the control group (*P <* *0.05, **P <* *0.01, ***P <* *0.001). Inhibition of the ERK1/2, p38 and JNK pathways in MDSCs treated with NPWT by the addition of PD98059, SB203580 and SP600125, respectively, showed a decreased in Runx2, COL‐I, OST and OPN gene expression at all time‐points (*P <* *0.05, **P <* *0.01, ***P <* *0.001).

**Figure 5:**
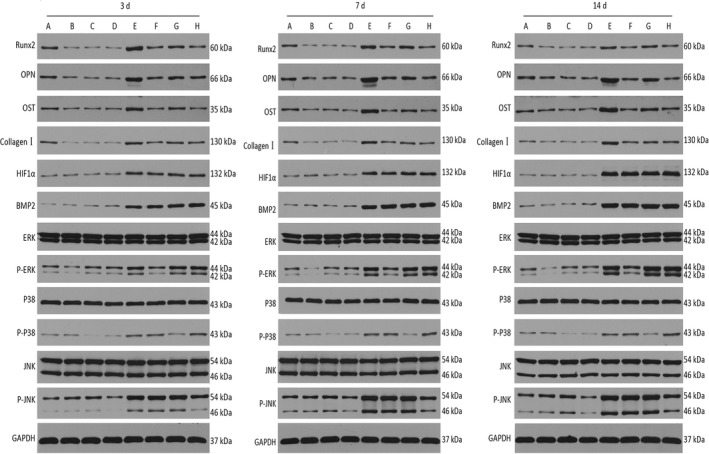
Western blot analysis. A (Ctr), B (Ctr+PD98059), C (Ctr+SB203580), D (Ctr+SP600125), E (NPWT), F (NPWT+PD98059), G (NPWT+SB203580) and H (NPWT+SP600125). HIF‐1α, BMP‐2, Runx2, COL‐I, OST and OPN protein expression in MDSCs treated with NPWT on day 3, 7 and 14 incubation in the presence of the inhibitors of the ERK1/2, p38 MAPK and JNK pathways. The Runx2, COL‐I, OST and OPN protein expression levels were decreased on all pathways, but the HIF‐1α, BMP‐2 protein expression levels did not demonstrate a significant effect on these pathways (*P <* *0.05, **P <* *0.01, ***P <* *0.001).

In the NPWT group, the Runx2 mRNA expression showed an initial increase (Fig. S2D) that peaked at day 7; then, there was a slight decline (Fig. 4). The mRNA expression levels of ALP, COL‐I, OST, OPN, HIF‐1α and BMP‐2 increased from day 3 to day 14 (Fig. 4). The Western blot analyses were in accordance with the RT‐PCR results (Fig. 5).

### Effects of specific inhibitors on osteogenic differentiation

ERK1/2, p38 and JNK pathway‐specific inhibitors were used to understand the effect on osteogenic differentiation. As shown in Figure 3A, we found that inhibition of the ERK1/2, p38 and JNK pathways led to a decreased trend compared to NPWT group. The results of the IOD analysis were in accordance with the alizarin red staining (Fig. S1). Inhibition of the ERK1/2, p38 and JNK pathways caused a decrease tendency in ALP activity in MDSCs treated with NPWT (Fig. 3B). MDSCs treated with NPWT showed a decrease in Runx2, COL‐I, OST and OPN gene expression at all time‐points by the addition of PD98059 (25 μM), SB203580 (10 μM) or SP600125 (25 μM) (Fig. 4). HIF‐1α and BMP‐2 gene expression did not show an effect by inhibition of the ERK1/2 pathway in NPWT‐treated MDSCs (Fig. 4A and D), although the Runx2, COL‐I, OST and OPN gene expression showed a decrease (Fig. 4B–F). Inhibition of the p38 pathway by the addition of SB203580 to NPWT‐treated MDSCs did not show a relevant effect on BMP‐2 (Fig. 4D) or HIF‐1α on day 3 (Fig. S2A) and the expression of the other genes decreased (Fig. S2B–F). Inhibition of the JNK pathway by the addition of SP600125 to NPWT‐treated MDSCs showed a significant effect on the expression of all genes (Fig. 4) except BMP‐2 on day 7 (Fig. 4F). The Western blot assay also showed that the levels of Runx2, COL‐I, OST and OPN on day 3 and day 7 were significantly down‐regulated in the presence of the inhibitors to the ERK1/2, p38 and JNK pathways when compared with the NPWT group (Fig. 5). These experiments revealed that NPWT promoted MDSC osteogenic differentiation through the MAPK pathway.

## Discussion

It is universally acknowledged that NPWT is a successful and useful therapeutic method for traumatic wounds. Many clinical reports have been published on its application in treatment of open fractures. However, the underlying mechanisms are less obvious. In this research, we provide new evidence that NPWT promotes osteogenic differentiation of MDSCs perhaps *via* the MAPK signalling pathway.

Fracture healing is a complicated process that depends upon various cells and factors. The osteocompetent progenitors originating from the periosteum and bone marrow play an important role during the process of bone repair 21, 22. However, open fractures caused by high energy often lead to periosteum and soft tissue damage, which have a much higher probability of non‐union or delayed union. In addition to the above cells involved in fracture healing, Liu *et al*. 11 reported that approximately 40% of the cells in open fractures with periosteal stripping were MDSCs. Therefore, we suggested that MDSCs might be considered to be one of the key cell types during the process of open fracture healing.

To the best of our knowledge, cell proliferation is the pivotal step of wound healing. Therefore, the influences of NPWT on MDSCs needed further investigation. McNulty AK *et al*. 23 reported that NPWT‐treated cells showed significantly greater cell proliferation than cells under static conditions. However, the apoptosis rate showed no obvious distinction between these two groups. Other studies 17, 24, 25 also showed that NPWT could promote cell proliferation and that cell proliferation was caused by micromechanical deformation produced by foam and continuous suction. Consistent with this research, our present study demonstrated that NPWT also promoted the proliferation of MDSCs at day 3 under sustained subatmospheric pressure. Compared with static conditions, the cell apoptosis rate was slightly increased in the MDSCs treated with NPWT, although there was no statistically significant difference between these two groups at day 3. Therefore, these results illustrated that NPWT could promote MDSC proliferation, and there was no remarkable effect on MDSC apoptosis.

Osteogenic differentiation plays a vital role in fracture healing. To further investigate whether NPWT could promote osteogenic differentiation of MDSCs *in vitro*, we examined the expression of osteogenic markers. Related articles showed that NPWT could promote the healing of open fractures in clinical and experimental studies 9, 26. In our previous research, we also found that NPWT could promote MSC osteogenic differentiation 17. In the present study, our results exhibited that ALP activity expression and mineralization were elevated in MDSCs treated with NPWT compared to the control group. Furthermore, the osteogenic gene and protein expression also showed the same results when MDSCs were treated with NPWT. However, Runx2 initially increased on day 3 and reached its peak on the seventh day before declining. These results were in accordance with a previous study 27 and were contrary to the reports of the effect of continuous mechanical strain stimulation on osteogenic differentiation of MSCs 28. In conjunction with our previous study 17, we suggested that mechanical stretch and hydrostatic pressure have a direct effect on the osteogenic differentiation of MDSCs. In addition, through preliminary experiments 17, we found that the cells in the fibrin matrix might be more sensitive to fluid shear stress, which might play a dominant role in mechanical stimulation by NPWT. Therefore, we concluded that NPWT could promote MDSC osteogenic differentiation.

Regional hypoxia is one of the principal mechanisms of NPWT. HIF‐1α was up‐regulated under the condition of hypoxia. A hypoxic environment could create an osteogenic favourable microenvironment and thus maintain the survival of osteoblasts 29. A previous study demonstrated that the expression of BMP‐2 was elevated under a low oxygen environment by activation of multiple signalling pathways, which includes MAPK signal pathways 30. Payne KA *et al*. 15 confirmed that MAPK pathways participate in osteogenic differentiation of MDSCs induced by BMP‐4 *in vitro*. Furthermore, many studies have exhibited that the MAPK family is activated by a variety of external stimuli 31, 32, 33, 34. With these external stimuli, BMP plays a major role in osteogenic differentiation. In our study, the ERK1/2, p38 and JNK pathways also play a pivotal role in osteogenic differentiation of MDSCs treated with NPWT. Our results showed that the ALP activity and mineralization were decreased in MDSCs treated with NPWT using the specific chemical inhibitors to the ERK1/2, p38 and JNK pathways, PD98059 (25 μM), SB203580 (10 μM) and SP600125 (25 μM), respectively. The osteogenic gene and protein expression also showed the same results when the NPWT was added into the MAPK pathway‐specific inhibitor. Previous research has shown that the MAPK signalling pathway was involved in the MDSC osteogenic differentiation 15. In their research, they thought that the ALP activity and mineralization were increased when the ERK1/2 pathway was inhibited, whereas inhibition of the p38 pathway decreased osteogenesis in the BMP4‐induced MDSCs. In the pluripotent stem cell C2C12 myoblast line, BMP‐2 has been shown to activate ERK1/2 and p38, but not to activate JNK 35. However, others reported that BMP‐2 primary activation of p38 and JNK in MC3T3‐E1 and calvaria‐derived osteoblastic cells, whereas BMP‐2 barely affects the activation of ERK1/2 16. We suggested that two reasons might lead to these differences.

The mechanism of NPWT is very complex, including mechanical stimulation, regional hypoxia and other mechanisms 36, 37. Previous articles have indicated that mechanical stimulation could promote osteogenic differentiation of MDSCs and MSCs in response to shear stress, and the ERK1/2, nitric oxide and p38, Ca^2+^ signalling pathways were activated 27, 38. On the other hand, as a previous study demonstrated, because of cross‐talk between these pathways, specific chemical inhibitors might affect multiple pathways. Furthermore, many studies have revealed that the ERK1/2, p38 and JNK pathway might be associated with BMP‐activated Smads 32, 39, 40. Therefore, we propose that the mechanism by which NPWT promotes osteogenic differentiation of MDSCs is rather complex. A large number of signals and factors might be involved in this process. The detailed mechanism needs further study. We would establish in an open fracture animal model to confirm the role of NPWT in bone healing *in vivo* for further research.

We demonstrated that NPWT could promote MDSC proliferation and osteogenic differentiation through experiments in this article. We found that osteogenic differentiation of MDSCs treated with NPWT was influenced by the addition of MAPK pathway‐specific inhibitors. The results showed the influence of NPWT on MDSC osteogenic differentiation *via* the MAPK pathway. We hope this study might provide a scientific basis to prove the positive role of NPWT in open fracture or bone defects.

## Declaration

This manuscript has never been partly or wholly published in any other journals. It is not being submitted to any other journal.

## Conflict of interest

The authors confirm that there are no conflict of interests.

## Supporting information


**Figure S1** The IOD analysis.
**Figure S2** RT‐PCR analysis on day 3.
