# Supplementary material for: Negative pressure wound therapy promotes muscle‐derived stem cell osteogenic differentiation through MAPK pathway
Source: J Cell Mol Med. 2017 Sep 25;22(1):511–20. doi: 10.1111/jcmm.13339 (PMC5742679; doi:10.1111/jcmm.13339)
Supplement: Supplementary file 1 — Figure S1 The IOD analysis. Figure S2 RT‐PCR analysis on day 3. [file JCMM-22-511-s001.docx]

**Supporting information**


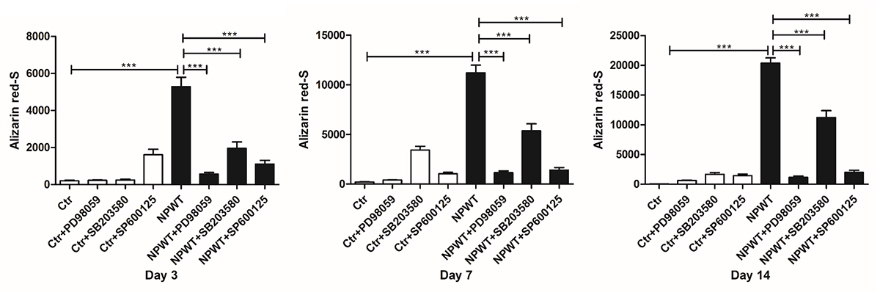


**Fig.S1 The IOD analysis.** The IOD analysis of MDSCs treated with NPWT and inhibitors to the ERK1/2, p38 MAPK, and JNK pathways on day 3, 7 and 14 showed a significant decrease compared to NPWT group (****p* <0.001).


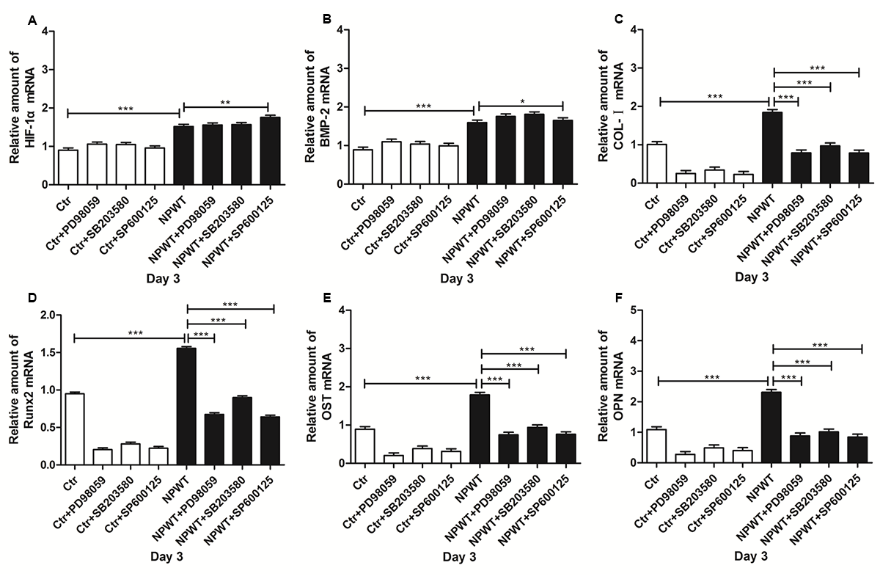


**Fig.S2** **RT-PCR analysis** **on day 3**. HIF-1α, BMP-2, Runx2, COL-I, OST and OPN gene expression in MDSCs treated with NPWT on day 3 incubation in the presence of the inhibitors to the ERK1/2, p38 MAPK, and JNK pathways. The expressions of the specific genes (**A**) HIF-1α, (**B**) BMP-2, (**C**) COL-I, (**D**) Runx2, (**E**) OST and (**F**) OPN were higher in NPWT group than those in control group (**p*<0.05, ***p*<0.01, ****p*<0.01). Inhibition of the ERK1/2, p38 MAPK, and JNK pathways to MDSCs treated with NPWT by addition of PD98059, SB203580, and SP600125 respectively, showed a decrease in Runx2, COL-I, OST and OPN gene expression at all time points (**p*<0.05, ***p*<0.01, ****p*<0.001).
